# Patient-reported outcomes as predictors of 10-year survival in women after acute myocardial infarction

**DOI:** 10.1186/1477-7525-8-140

**Published:** 2010-11-25

**Authors:** Tone M Norekvål, Bengt Fridlund, Berit Rokne, Leidulf Segadal, Tore Wentzel-Larsen, Jan Erik Nordrehaug

**Affiliations:** 1 Department of Heart Disease, Haukeland University Hospital, Bergen, Norway; 2 Department of Public Health and Primary Health Care, University of Bergen, Bergen, Norway; 3 School of Health Sciences, Jönköping University, Jönköping, Sweden; 4 Department of Surgical Sciences, University of Bergen, Bergen, Norway; 5 Centre for Clinical Research, Haukeland University Hospital, Bergen, Norway; 6 Institute of Medicine, University of Bergen, Bergen, Norway

## Abstract

**Background:**

Patient-reported outcomes are increasingly seen as complementary to biomedical measures. However, their prognostic importance has yet to be established, particularly in female long-term myocardial infarction (MI) survivors. We aimed to determine whether 10-year survival in older women after MI relates to patient-reported outcomes, and to compare their survival with that of the general female population.

**Methods:**

We included all women aged 60-80 years suffering MI during 1992-1997, and treated at one university hospital in Norway. In 1998, 145 (60% of those alive) completed a questionnaire package including socio-demographics, the Sense of Coherence Scale (SOC-29), the World Health Organization Quality of Life Instrument Abbreviated (WHOQOL-BREF) and an item on positive effects of illness. Clinical information was based on self-reports and hospital medical records data. We obtained complete data on vital status.

**Results:**

The all-cause mortality rate during the 1998-2008 follow-up of all patients was 41%. In adjusted analysis, the conventional predictors s-creatinine (HR 1.26 per 10% increase) and left ventricular ejection fraction below 30% (HR 27.38), as well as patient-reported outcomes like living alone (HR 6.24), dissatisfaction with self-rated health (HR 6.26), impaired psychological quality of life (HR 0.60 per 10 points difference), and experience of positive effects of illness (HR 6.30), predicted all-cause death. Major adverse cardiac and cerebral events were also significantly associated with both conventional predictors and patient-reported outcomes. Sense of coherence did not predict adverse events. Finally, 10-year survival was not significantly different from that of the general female population.

**Conclusion:**

Patient-reported outcomes have long-term prognostic importance, and should be taken into account when planning aftercare of low-risk older female MI patients.

## Background

Research on long-term survival after acute myocardial infarction (MI) in older women is scarce. Characteristically, the population-based MONICA-studies [1] had an age limit of 64 years. Similarly, few studies have investigated patient-reported outcomes in female long-term MI survivors.

There is a growing recognition of the importance of a patient perspective on health after medical treatment of cardiovascular disease [2,3]. Patient-reported outcomes can provide an additional measure complementary to objective biomedical measures. One interesting question is whether the patients' own experience of health and quality of life (QOL) has prognostic importance.

In their early review of 27 community studies, Idler & Benyamini [4] found that global self-rated health (SRH) was an independent predictor of mortality, despite the inclusion of relevant covariates known to predict mortality. In the majority of studies the association was stronger for men. However, more recent studies have shown contradictory results [5]. With respect to patients with acute MI, studies have focused on patient-reported outcomes in relation to short-term mortality [6,7], have mainly included male patients [7-10] or patients below 70 years of age [7,9-11]. Concerning QOL, an association with mortality has been reported [7,11], although diverse use of the concept makes comparison between studies difficult. Most studies, however, have focused on the role of negative emotions on outcome in cardiac disease [12]. Applying a salutogenic approach by investigating other patient-reported outcomes, like sense of coherence (SOC) [13] and perceived positive effects of illness [14,15], has thus far shown mixed results in predicting adverse events [16,17], but is proposed to have a potential protective effect [18].

We included in our study women 60-80 years who had at least 3 months post MI and were in a clinically stable condition. The primary aim was to determine whether 10-year survival in older women after MI is related to SRH and other patient-reported outcomes; QOL, SOC and perceived positive effects of illness. A secondary aim was to compare the survival of such older female MI survivors with the general population matched for age, gender and time.

## Methods

### Design and setting

A prospective design was applied including all women with MI treated at one university hospital during a 5-year period. Clinical variables were recorded from index infarction (1992-1997); self-reported questionnaires were completed 3 months to 5 years after MI (1998); and all patients were followed up for 10 years (until 2008). Informed consent was obtained from the subjects [19], and the study was approved by the Regional Committee for Medical Research Ethics, Western Norway, and the Norwegian Social Science Data Services.

### Study participants

**Figure 1:**
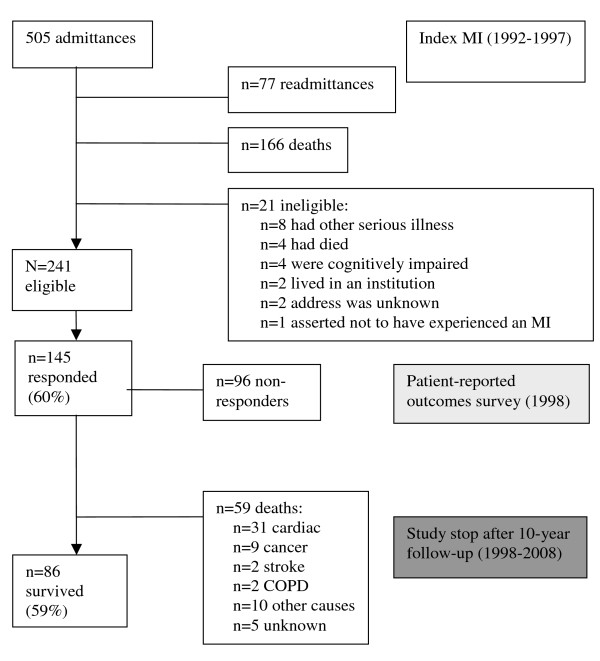
**Flow chart of the sampling and timeframe of the study**.

The study inclusion criteria comprised the total population of women aged 60-80 years, hospitalized within a 5-year period (1992-1997), diagnosed with MI (ICD-9 CM code 410), and now living at home. Having other serious illness like cancer or stroke, or being cognitively impaired, disqualified subjects from participating. A detailed description of the sampling is presented in Figure 1. A total of 145 women (60%) returned the questionnaire and were available for the present prospective study. The responders did not differ significantly from those not responding to the survey with regard to age (mean 72.0 vs. 72.8 years, p = 0.154); time since MI (mean 29 vs. 31 months, p = 0.496); or length of hospital stay (mean 9 vs. 10 days, p = 0.364).

### Measurements

Socio-demographic and clinical variables were included as shown in Table 1. MI was defined according to the WHO [20] (for events in 1992-2000) and ESC/ACC [21] (for events in 2001 and onwards). Left ventricular ejection fraction (EF) was determined by echocardiography.

**Table 1:** Socio-demographic and clinical characteristics, and hazard ratios for MACCE and all-cause mortality (N = 145).

			MACCE n = 52		All-cause mortality n = 59	
	n*	%	HR	p-value	HR	p-value
**Socio-demographics:**						
**Mean age in years (SD)**	72 (5)		1.05	0.131	1.06	**0.044**
**Cohabitation status**				**0.007**		**<0.001**
- Living alone	60	41	2.12		2.87	
- Cohabitation	85	59	(ref)		(ref)	
**Marital status**				**0.003**		**0.009**
- Divorced	7	5	4.57	**0.007**	3.20	**0.036**
- Widowed	62	43	2.76	**0.001**	2.61	**0.001**
- Unmarried	6	4	0.84	0.868	2.71	0.111
- Married	68	48	(ref)		(ref)	
**Educational status**				0.085		0.098
- Elementary school	61	44			(ref)	
- Secondary school	41	29	1.09	0.804	1.29	0.441
- High school and university/college	37	27	2.00	**0.039**	1.99	**0.032**
**Clinical characteristics:**						
**Risk factors of CAD**						
- Mean total cholesterol, mmol/L (SD)	7.0 (1.4)		1.10	0.400	1.05	0.660
- Hypertension	53	37	0.96	0.877	1.30	0.318
- Diabetes mellitus	17	12	1.74	0.130	1.22	0.607
- Overweight	42	39	0.90	0.740	1.14	0.665
- Family history of CAD	59	68	1.85	0.152	2.06	0.115
- Smoking habits				0.531		0.817
- Non smoker	68	55	1.24	0.528	1.16	0.674
- Ex-smoker	21	17	0.77	0.607	1.31	0.528
- Current smoker	34	28	(ref)		(ref)	
**Previous angina**	62	45	1.27	0.397	1.23	0.441
**Previous acute MI**	32	23	1.09	0.788	1.11	0.734
**Mean time since MI in months (SD)^† ^**	29 (16)		1.01	0.333	1.01	0.398
**Disease severity**						
- Mean max CK (SD)	1099 (1000)		1.00	0.540	1.00	0.744
- Q in ECG	63	44	0.83	0.502	1.22	0.447
- Left ventricular ejection fraction				0.108		**0.012**
- >60%	78	62	(ref)		(ref)	
- 30-60%	45	36	0.97	0.926	1.19	**0.568**
- <30%	2	2	4.69	**0.038**	9.88	**0.003**
**Mean creatinine, μmol/L (SD)^‡^**	92.5 (18.9)		1.07	0.386	1.18	**0.028**
**Treatment during index MI**				0.258		0.793
- Medical treatment	92	66	0.43	0.164	1.99	0.497
- Thrombolysis	43	31	0.35	0.100	1.95	0.517
- PCI/CABG	4	3	(ref)		(ref)	

**Medication at discharge after index MI**						
- Beta blockers	98	69	0.50	**0.015**	0.77	0.340
- Calcium antagonists	18	13	0.47	0.199	1.11	0.789
- ACE inhibitors	40	28	1.44	0.232	1.36	0.281
- Diuretics	48	34	1.60	0.109	1.66	0.060
- Digitalis	9	6	1.97	0.152	1.19	0.738
- Antithrombotics	123	86	1.04	0.924	1.14	0.730
- Lipid-lowering	26	18	1.05	0.899	0.71	0.360
- Antidiabetics	12	8	1.96	0.100	1.03	0.955

Significant results are shown in bold.

*n varies between the different variables because of missing values. ^†^Time from index MI to survey. ^‡^Logtransformed as independent variable, HR per 10% increase.

MACCE, major adverse cardiac and cerebral events; CAD, coronary artery disease; CK, creatinine kinase; PCI, percutaneous coronary intervention; CABG, coronary artery bypass grafting.

To measure QOL, we used the World Health Organization Quality of Life Instrument Abbreviated (WHOQOL-BREF), which contains 26 items and four domains: physical health, psychological, social relationships, and environmental domain. A profile of domain scores is generated, scaled from 0 to 100, with higher scores denoting higher QOL. Scoring was performed according to the manual [22]. Investigation of missing data in this dataset was reported in detail elsewhere [19]. WHOQOL-BREF has been shown to be valid and reliable in other studies, although the social domain has represented a challenge [23]. In the present study, internal consistency (Cronbach's alpha) ranged from 0.58 for the social domain to 0.82-0.83 for the other domains. WHOQOL-BREF also includes two global items on overall QOL and SRH, rated on a 5-point Likert scale. In the survival analysis we merged the "poor" and "very poor" response categories for overall QOL. For SRH we merged the "very dissatisfied" and "dissatisfied" categories, and the "very satisfied" and "satisfied" categories.

Symptoms and function were assessed by using five questions scored from 1 to 5, including perceived chest pain, perceived insecurity about physical exercise, thinking about the illness, ability to walk 2 kilometers, and coronary artery disease (CAD) affecting daily activities. An index was computed on a scale of 0-100, such that higher scores denote fewer symptoms and higher function. Participants had to respond to at least 3 of 5 items in order for a summary score to be obtained. Cronbach's alpha was 0.71.

A single-item question on possible positive effects of illness was used: "*All in all, was there anything positive about experiencing an MI?" *Potential subjects were instructed to answer "yes" or "no" to this item [15].

The sense of coherence scale (SOC-29) measures coping capacity by using 29 items, scaled from 1 to 7 with two anchors, and has a possible total score of 29-203. Higher scores indicate a stronger SOC [13]. Details on handling of missing scores were described previously [24]. SOC-29 has proven to be valid and reliable [25]. In the present study, Cronbach's alpha was 0.93.

### Data collection

Patient reports were obtained by postal questionnaires distributed to all candidate subjects satisfying the inclusion criteria regardless of type of follow-up, or whether any intervention had taken place, and who in December 1997 were alive as determined by the hospital patient administration system and the National Population Register of Statistics Norway. Non-responders were reminded once. Questionnaires were returned by February 27, 1998, and all patients were followed up for 10 years (February 27, 2008), or until death. Information on mortality rates of the Norwegian general population was made available through Statistics Norway.

### Classification of events during follow-up

Endpoints were all-cause death and major adverse cardiac and cerebral events (MACCE). MACCE was defined as a composite of cardiac death, non-fatal MI, and stroke. Events were recorded from the date of return of the questionnaires. The International Classification of diseases (ICD) version 9 was used when including patients into the study and to identify readmissions during follow-up in 1998, and version 10 was used from 1999 onwards.

Survival status was determined 10 years after the questionnaires were returned, and up to 15 years since index MI, through the National Population Register of Statistics Norway by means of a unique personal identification number. For patients dying in hospital (n = 26; 44% of all deaths), the cause of death was classified on the basis of diagnosis and discharge notes. The cause of death of patients dying out of hospital was based on an assessment of discharge notes and diagnosis of the two last hospitalisations of the patient. All re-admissions and in-hospital deaths were tracked through the hospital information system and verified by reviewing all patient medical records. The underlying cause of death (the disease or injury that initiated the cascade of morbid events resulting in death) was defined as the cause of death. Sudden death and death not attributable to non-cardiac disease were classified as cardiac deaths. Non-cardiac death consisted of cancer, stroke, chronic obstructive pulmonary disease, and one group classified as 'other causes of death'.

### Statistical analysis

Survival analyses with 'time since survey' as time variable were performed by the Kaplan-Meier procedure with log-rank tests. Survival was compared with the general population, matched for age, gender and calendar year by use of the so-called direct method [26]. Mortality rates in 1-year intervals were used (Statistics Norway).

Hazard ratios (HR) with 95% confidence intervals (CI) were computed based on univariate and multivariate Cox regression analysis using socio-demographic, clinical and patient-reported outcomes as predictors with time to MACCE and all-cause mortality as endpoints. Predictive models were developed on the basis of previous research and our clinical experience. The distribution of serum creatinine was markedly skewed and therefore this variable was logarithmically transformed. The proportional hazard assumptions in the multivariate Cox regression analyses were checked as recommended by Therneau and Grambsch [27]. All tests were two tailed, with a level of significance set at p≤0.05. Comparison with the general population was performed using an application locally developed in Visual Basic for Windows (Microsoft 2003). The investigation of Cox assumptions used the package survival in R (The R Foundation for Statistical Computing, Vienna, Austria). All other analyses were performed with SPSS 15 (SPSS Inc, IL, USA).

## Results

**Figure 2:**
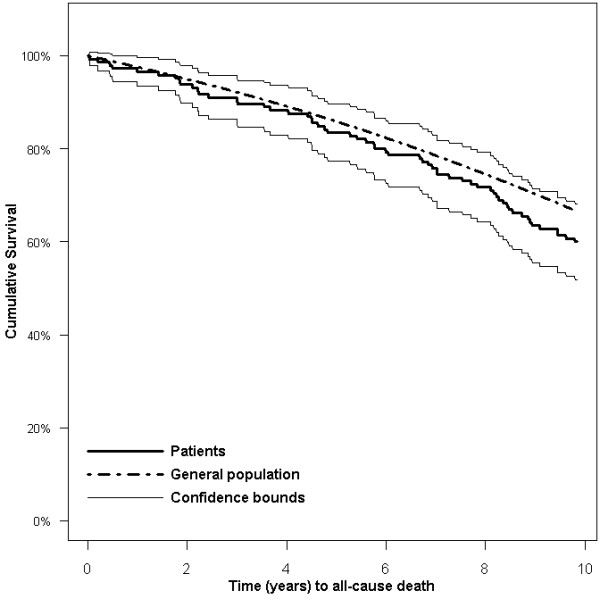
**Survival in older women 10 years after survey (up to 15 years after MI) compared to expected survival based on the Norwegian general population matched for age, gender, and time**.

Of the 145 participants included in this prospective follow-up study, 59 (41%) had died after 10 years. Thirty-one (57%) died from cardiac causes, nine from cancer, two from stroke, two from chronic obstructive pulmonary disease, and 10 from other causes. Vital status for all patients was complete, although the cause of death of five patients could not be determined (Figure 1). When compared with women in the general population matched for age and calendar year, the survival of these older women did not differ significantly from survival of women in the general population (Figure 2). The relative survival was not at any point in time lower than 90%.

### Patient characteristics

The mean age in this female MI cohort was 72 years (range 62-80 years), and 41% were living alone. The majority of those living with someone lived with a spouse or partner (85%), whereas 12% lived with their children. Time since index MI ranged from 3 months to 5 years. Mean serum creatinine was 92.5 μmol/L, 38% of the MI survivors had a reduced EF, and 12% were diagnosed with diabetes. Patient characteristics are presented in further detail in Table 1. Descriptive summaries of patient-reported outcomes (SRH, QOL variables, SOC and perceived positive effects) are included in Table 2.

**Table 2:** Patient-reported outcomes, and hazard ratios for MACCE and all-cause mortality (N = 145).

			MACCE n = 52		All-cause mortality n = 59	
	n*	%	HR	p-value	HR	p-value
**Quality of life domains, mean (SD)**						
- physical health domain,	57 (18)		0.99	0.897	0.90	0.142
- psychological domain	67 (15)		0.95	0.594	0.94	0.430
- social relationships domain	71 (16)		0.93	0.443	1.04	0.672
- environmental domain	64 (16)		0.99	0.879	0.99	0.861
**Overall quality of life**				0.795		0.328
- very poor/poor	9	6		(ref)		(ref)
- neither poor nor good	38	27	0.91	0.885	0.74	0.560
- good	75	53	0.73	0.599	0.57	0.251
- very good	20	14	0.61	0.484	0.36	0.103
**Self-rated health**				0.531		0.073
- dissatisfied/very dissatisfied	22	15	1.37	0.433	2.12	**0.027**
- neither satisfied nor dissatisfied	48	33	0.84	0.583	1.10	0.765
- satisfied/very satisfied	73	50		(ref)		(ref)
**Symptoms and function, mean (SD)**	62 (24)		0.99	0.837	0.94	0.308
**Positive effects of illness**				0.075		**0.021**
- yes	87	65	1.86		2.14	(ref)
- no	47	35		(ref)		
**Sense of coherence, mean (SD)**	144 (26)		0.97	0.623	1.00	0.991

*n varies between the different variables because of missing values.

Significant results are shown in bold.

Hazard ratios for WHOQOL-BREF subscales, symptoms and function and sense of coherence are per 10 points differences.

MACCE, major adverse cardiac and cerebral events.

### Univariate predictors of outcome

**Figure 3:**
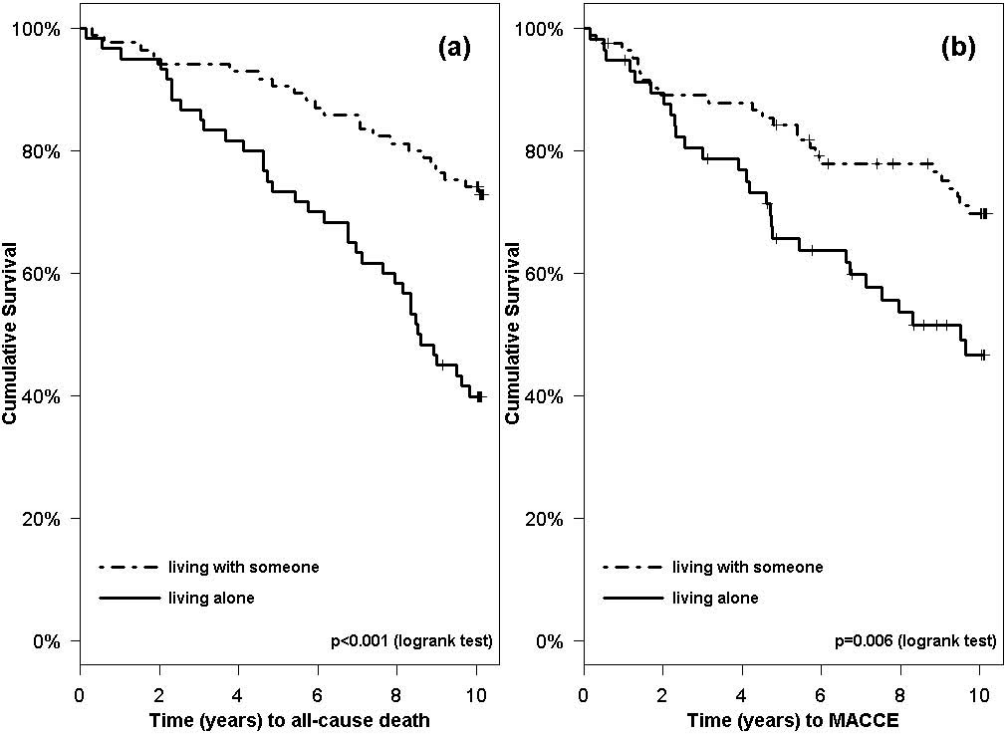
**Kaplan-Meier curves on time to (a) all-cause death and (b) MACCE in women after MI, living alone vs living with someone**.

Women living alone had a significantly increased all-cause mortality and risk of MACCE compared to those living with someone. Kaplan-Meier curves for cohabitation in relation to all-cause mortality and time to MACCE are shown in Figure 3. Among the clinical indicators, creatinine level and reduced EF significantly predicted all-cause mortality. Use of beta blockers was associated with lower occurrence of MACCE. Time from index MI to inclusion was not related to all-cause mortality or MACCE (Table 1).

As shown in Table 2, those dissatisfied with their general health had a two times higher risk of dying compared to those satisfied with their general health. Other patient-reported outcomes did not predict MACCE or all-cause death, except perceived positive effects of experiencing an MI. Those reporting positive effects had significantly higher risk of all-cause death than those that did not. However, this was not the case for MACCE.

### Multivariable prognostic models

**Figure 4:**
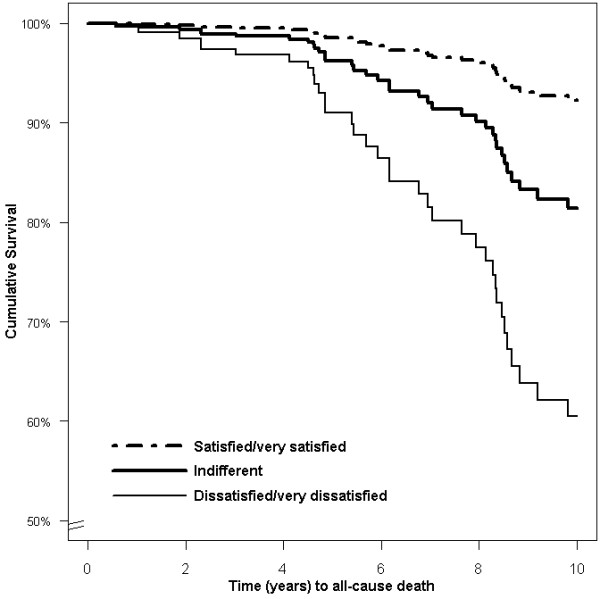
**Survival in women after MI in relation to self-reported health**. Multivariate Cox regression with data based on a typical cohabiting, 70-year-old woman with creatinine of 90 μmol/L, left ventricular ejection fraction >60%, average scores on sense of coherence and quality of life domains, and who perceived positive effects of MI.

Multivariable Cox regression analysis for overall survival was performed that included selected socio-demographic, clinical, and patient-reported variables, the results of which are shown in Table 3. Living with someone, higher satisfaction with SRH (as shown in Figure 4), higher scores on psychological and lower on environmental QOL domain, higher EF, lower creatinine levels, and not perceiving positive effects of illness were positively related to overall survival, whilst scores on the physical health domain, social relationships domain, and SOC were not. In the MACCE model, we found living alone, diabetes, and lower EF, along with lower scores on two of the QOL domains and perceiving positive effects of illness, to be significant predictors of adverse events. There were no indications of deviations from the the Cox proportional hazard assumptions (global p = 0.621 for overall survival and 0.166 for MACCE).

**Table 3:** Multivariate Cox regression analysis of risk factors for MACCE and all-cause mortality in older women after MI (N = 145).

Predictor variables	MACCE n = 52			All-cause mortality n = 59		
	HR	CI	p-value	HR	CI	p-value
**Socio-demographics:**						
Cohabitation status			**<0.001**			**<0.001**
- Living alone	6.07	(2.69-13.69)		6.24	(2.68-14.51)	
- Cohabitation	(ref)			(ref)		
**Conventional predictors:**						
Creatinine				1.26	(1.01-1.56)	**0.041**
Diabetes mellitus	3.89	(1.29-11.73)	**0.016**			
Left ventricular ejection fraction			**0.023**			**0.004**
- >60%	(ref)			(ref)		0.236
- 30-60%	0.82	(0.39-1.74)	0.604	0.60	(0.26-1.40)	0.236
- <30%	11.12	(1.86-66.52)	**0.008**	27.38	(3.18-235.76)	**0.003**
**Patient-report:**						
Physical health domain	1.17	(0.89-1.55)	0.267	1.13	(0.88-1.46)	0.322
Psychological domain	0.64	(0.43-0.95)	**0.026**	0.60	(0.40-0.90)	**0.015**
Social relationships domain	0.67	(0.50-0.92)	**0.012**	1.37	(0.90-2.09)	0.144
Environmental domain	1.77	(1.24-2.53)	**0.002**	1.90	(1.30-2.77)	**0.001**
Self-rated health			0.209			**0.028**
- dissatisfied/very dissatisfied	2.44	(0.59-10.12)	0.220	6.26	(1.63-24.01)	**0.007**
- neither satisfied nor dissatisfied	0.77	(0.28-2.10)	0.605	2.56	(0.86-7.57)	0.090
- satisfied/very satisfied	(ref)			(ref)		
Positive effects of illness			**0.001**			**0.001**
- yes	5.13	(1.88-14.02)		6.30	(2.22-17.83)	
- no	(ref)			(ref)		

Sense of coherence	1.02	(0.82-1.27)	0.850	1.05	(0.83-1.32)	0.692

Adjusted for age and time since MI. Significant results are shown in bold.

MACCE, major adverse cardiac and cerebral events.

Hazard ratios for WHOQOL-BREF subscales and sence of coherence are per 10 points differences, for creatinine per 10% increase.

## Discussion

Using well-established questionnaires, we examined the relationship between patient-reported outcomes and long-term survival in women after MI. We also compared the survival of our cohort with that of the general population, matched for age, gender and time. We found that women living alone had significantly increased risk of MACCE and all-cause death. Patient-reported outcomes like higher scores on SRH and the psychological QOL domain, as well as higher EF and lower creatinine levels, were positively related to overall survival. The presence of diabetes, lower EF, lower scores on psychosocial QOL domains, and experience of positive effects of illness predicted MACCE. Survival in this female MI cohort was not significantly different from that of the general population.

During the last decades, survival after MI has improved, mirroring the improvements in risk-factor management, pharmacological treatment, and revascularization techniques [28]. Studies using landmark analysis have shown that survival benefit levels off in the long-term. However, the fact that survival in this selected cohort was not different from that of the general population is remarkable, considering that these women did not receive what today is recommended as full secondary prevention [29]. In particular, lipid-lowering therapy was scarce in this cohort. On the other hand it is important to note that the majority of patients were non-smokers and received anti-thrombotics and beta-blockers (Table 1). Furthermore, this cohort is a low risk MI population as 41% died before inclusion into the study. We thereby avoided the impact of strong clinical predictors on short-term post-infarction mortality, like reinfarction after thrombolytic therapy, ventricular arrhythmias, and poor left ventricular function. The final balance of all these factors may explain our results on this point.

Living alone was clearly a risk factor for both MACCE and all-cause death in women after MI. A few early studies have reported that living arrangements affect mortality post MI [30,31]. Since then, the protective effect of living with someone has been reported by several studies [32]; however, in cardiac populations, this effect has mainly been shown in men [33]. As patients living alone are more likely to be older women, our study findings contribute important information. Living alone may be seen as an indicator of social isolation, which tends to be associated with higher risk behaviours [34], and perhaps also poorer adherence to medication and other follow-up recommendations. However, living with someone has also been reported to have negative effects due to marital stress [35] and caregiving strain [36]. Given that some of our cohabiting women may have experienced some of these negative effects makes the results even more convincing. Hence, we recommend including patients' living arrangements in post-discharge care planning in order to optimize outcomes after MI. Peer support groups [37] and rehabilitation programmes [38] may offer valuable contributions. However, there are few randomized trials that have attempted to improve low social support. As a result, the impact on clinical endpoints is not known [39].

To the best of our knowledge, this study is the first to report on SRH as an independent predictor of long-term mortality in older women after MI. Women dissatisfied with their general health had more than six times higher risk of dying than those satisfied. Our findings support the recommendations of Krumholz et al. [3] to include SRH measurements into clinical practice in order to identify patients at high risk for adverse outcomes. A single measure of SRH can quite easily be obtained, and there is widespread agreement that SRH provides a useful summary of how people perceive their overall health status [40].

The psychological QOL domain predicted both MACCE and death from any cause. Previous investigation of this cohort demonstrated scores on the psychological QOL domain comparable to those of the general population [19]. The predictive power of this variable is therefore striking. However, another psychological measure, SOC, did not predict adverse events in women after MI. Not many studies have explored this line of research, but Surtees et al. [16] found a strong SOC to be significantly related to reduced cancer mortality in men. In line with our findings, this was not the case in women. Possibly, also length of follow-up may be of significance. A recent population based study showed that SOC predicted one-year mortality, but not 4-year mortality among very old people (aged 85-103 years) [41]. Another large population based study showed similar results; Finnish middle-aged men with weak SOC showed a higher mortality risk in an 8-year follow-up study [42], but this effect was weakened after 12 years [43]. No women were included in the study. The change in predictive power of SOC over time is interesting since SOC has been found to be a stable trait in the majority of studies, although some conflicting results have been reported [25]. In accordance with this, we also found SOC to be stable in another sub-study on this cohort [24]. However, it may well be that, although being a stable trait, SOC is important in the short term after critical illness, and that other factors are of more importance in the long run. In general, there is a possibility that the predictive value of variables decreases with time, as random events accumulate. However we found no indications for deviance from the Cox assumptions. The prognostic value of sense of coherence warrant further study, particularly in women.

We also found women reporting positive effects from experiencing an MI to have an increased risk of dying. This rather surprising finding is difficult to explain, although it has been suggested that positive affect in seriously ill populations can be associated with underreporting of symptoms, overoptimistic expectations, denial of seriousness of disease and failure to seek medical care or adhere to advice from health care professionals [17]. Consequently, high levels of positive affect could thereby be potentially harmful. Similar findings were reported in one frequently cited randomized trial on support of distressed MI patients, the M-HART trial [44], in which the intervention failed to protect against reinfarction, cardiac, or all-cause mortality in men, and had a possible harmful impact on women.

### Methodological issues

The strengths of this study are the employment of standardized and validated questionnaires targeting an understudied group of patients, the complete data on vital status and the 10-year follow-up of all subjects. The fact that 41% died before inclusion may have introduced a selection bias. Hence, our results can only be extrapolated to low-risk populations. The women had different time elapsed between index MI and inclusion, although this was not associated with adverse events in adjusted or unadjusted analyses. Furthermore, we had a 60% response rate to our survey. However, non-responders did not differ from responders on important variables, although differences in other unidentified confounders not accounted for cannot be excluded. A larger sample size would have allowed more variables to be included in the multivariate models.

## Conclusion

This study demonstrates that in female long-term MI survivors, the patients' personal experience, including living alone, has prognostic importance for long-term outcome after MI. SRH and certain QOL issues were important for longevity. Well-known factors, like renal function and left ventricular ejection fraction remained important and significantly predicted adverse outcome. Possible clinical implications include sensitivity to patient perceptions regarding the state of health and life situation as well as living arrangements when planning aftercare for older female MI patients. Further study is needed on patient-reported outcomes and their predictive power in women after MI.

## Abbreviations

EF: Left ventricular ejection fraction; MACCE: Major adverse cardiac and cerebral events; MI: Myocardial infarction; QOL: Quality of life; SOC: Sense of coherence; SOC-29: The sense of coherence scale; SRH: Self-rated health; WHOQOL-BREF: The World Health Organization Quality of Life Instrument Abbreviated;

## Competing interests

The authors declare that they have no competing interests.

## Authors' contributions

TMN designed the study, carried out the female MI survivor survey, collected all the patient data and drafted the manuscript. BF participated in the design of the study. JEN participated in the design of the study, and collection of medical records data by reviewing the ECGs and assessing cause of death. LS collected the yearly mortality rates of the general population and made the expected survival curves for the general population compared to study participants. TWL and TMN planned and performed all other data analysis. All authors commented on drafts of the manuscript, and read and approved the final manuscript.
